# Nationwide Inventory of Mosquitoes and the Distribution of Invasive *Aedes* (*Stegomyia*) *albopictus* (Skuse, 1894) on the Islands of Sao Tome and Principe in Central Africa

**DOI:** 10.3390/insects15080560

**Published:** 2024-07-23

**Authors:** Tsai-Ying Yen, Chien-Fu Cheng, Lien-Fen Tseng, Ronalg Mendes Costa d’ Assunção Carvalho, Kun-Hsien Tsai

**Affiliations:** 1 Institute of Environmental and Occupational Health Sciences, College of Public Health, National Taiwan University, Taipei 100025, Taiwan; yenty@cdc.gov.tw; 2 Center for Diagnostics and Vaccine Development, Centers for Disease Control, Ministry of Health and Welfare, Taipei 115201, Taiwan; 3 Taiwan Anti-Malaria Advisory Mission, Sao Tome, Sao Tome and Principe; again1118@gmail.com (C.-F.C.); titinatseng@gmail.com (L.-F.T.); 4 Taiwanese Medical Mission, Sao Tome, Sao Tome and Principe; ronalgcarvalho@hotmail.com; 5 Global Health Program, College of Public Health, National Taiwan University, Taipei 100025, Taiwan

**Keywords:** *Aedes albopictus*, the Democratic Republic of Sao Tome and Principe (DRSTP), Central Africa, cytochrome C oxidase subunit I (*COI*)

## Abstract

**Simple Summary:**

Mosquito surveys conducted in the Democratic Republic of Sao Tome and Principe during 2000 to 2016 as a part of anti-malaria programs found the presence of four species of mosquitoes in the nation including *Culex* (*Culex*) *poicilipes*, *Mansonia* (*Coquillettidia*) *annetti*, *Uranotaenia* (*Uranotaenia*) *alboabdominalis*, and *Uranotaenia* (*Uranotaenia*) *fraseri* for the first time. *Aedes albopictus* (Skuse, 1894) was identified in 2015, and the follow-up survey confirmed that *Aedes albopictus* has become widespread across the nation. The larvae were predominant in artificial water-holding containers, with a positive rate up to 45.6% in used tires in Príncipe, while the native species, *Aedes aegypti*, preferred natural breeding sources. Phylogenetic analysis based on mitochondrial DNA revealed the introduced populations belonged to a clade involved in the worldwide spread of the species. *Aedes albopictus* is a public health threat due to its vectorial capacity for various arboviruses. Continuous vector surveillance and implication of interventions, such as source reduction to remove used tires, environmental management, and use of larvicides, were suggested.

**Abstract:**

*Aedes albopictus* (Skuse, 1894), a mosquito originating in Asia, has been introduced to Africa since the 2000s. The mosquito is not only a nuisance but is capable of transmitting various arboviruses. The current study summarized our entomological surveys in the Democratic Republic of Sao Tome and Principe during 2000 to 2016. Adult mosquitoes were collected by sweep nets, human landing catches, and Centers for Disease Control (CDC) light traps, and the immatures were collected from water-filled habitats at 15 sentinel sites and reared to adulthood. Species identification was performed based on morphologic characteristics. Fragments of the cytochrome C oxidase subunit I (*COI*) and the *Wolbachia* surface protein (*wsp*) genes were amplified for mosquitoes collected in Principe. New records of four mosquito species were reported. *Aedes albopictus* was identified in 2015. The larvae were found distributed over the nation and were predominately in artificial water-holding containers (488/2698, 18.1%). The highest positive rate was observed in used tires in Príncipe (114/250, 45.6%). Mitochondrial DNA analysis revealed low genetic diversity among the invasive populations, but all tested specimens were superinfected by *Wolbachia*. The ability of *Ae. albopictus* to adapt to new environments and its involvement in disease transmission make the surveillance and control of this species particularly important.

## 1. Introduction

The emergence of the COVD-19 pandemic has sounded an alarm to the world about the threats of infectious diseases. Although Africa appeared to be less affected, vector-borne infectious diseases, such as malaria, still claim more than 550,000 lives each year [1]. Meanwhile, arboviral infections are becoming evident as public health concerns. Dengue virus (DENV) has been linked to at least 35 of 54 African countries [2,3]. Chikungunya outbreaks were reported in Gabon, Central Africa [4,5]. Zika virus (ZIKV), first identified in Uganda and dispersed across Asia, the Pacific, and Latin America, was shown to circulate in sub-Saharan Africa [6,7]. The worldwide spread of dengue, chikungunya, and Zika viruses is primarily attributed to *Aedes* (*Stegomyia*) *aegypti* (Linnaeus, 1762), but *Aedes* (*Stegomyia*) *albopictus* (Skuse, 1894) is a vector of increasing importance as well. Also called the Asian tiger mosquito, *Ae. albopictus* originates in Asia but has colonized on every continent except Antarctica with the aid of international trade and travel in the last three decades [8,9]. The first record of *Ae. albopictus* in Africa was in Cape Town, South Africa in 1989, but the invasive population was successfully controlled [10,11]. A few years later, *Ae. albopictus* was found in Nigeria, Cameroon, Equatorial Guinea, and Gabon [12]. The presence of *Ae. albopictus* on Sao Tome Island was not documented until 2017, while the mosquito was identified at four locations on the island [13]. *Aedes albopictus* was then recorded on Principe Island in limited sampling sites, with limited information about the ecological features and types of breeding sources [14,15].

**Figure 1:**
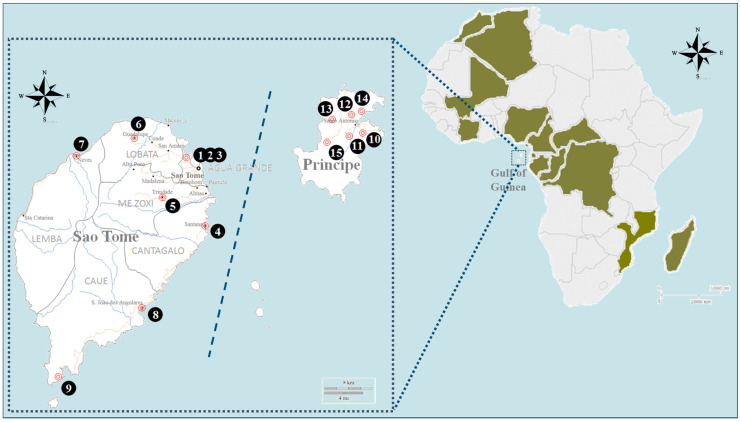
Sampling sites for *Aedes albopictus* in the Democratic Republic of Sao Tome and Principe (DRSTP). The continental African countries infested by *Ae. albopictus* are marked (green). 1. Ponte Graca; 2. Campo de Milho; 3. Boa Morte; 4. Santana; 5. Trindae; 6. Guadulupe; 7. Neves; 8. Sao Joao dos Angolares; 9. Porto Alegre; 10. Santo Cristo; 11. Porto Real; 12. Aeroporto; 13. Ponta do Sol; 14. Picao; 15. Sao Joaquim.

The Democratic Republic of Sao Tome and Principe (DRSTP) is an island nation in the Gulf of Guinea (Figure 1). The climate is tropical with an average annual temperature of approximately 27 °C. The rainy season lasts from October to May. Lying on the equator, the DRSTP forms part of the Central African tropical forest ecosystem, which is rich in mosquito fauna [14,16]. However, most ecological and genetic research have focused on *Anopheles* spp. because the nation used to suffer greatly from malaria [17]. An early survey has documented five species of *Aedes* mosquitoes on the islands including *Ae. aegypti*, *Aedes* (*Stegomyia*) *africanus*, *Aedes* (*Neomelaniconion*) *circumluteolus*, *Aedes* (*Aedimorphus*) *gandarai*, and *Aedes* (*Aedimorphus*) *nigricephalus* [16]. *Aedes* (*Catageomyia*) *tarsalis* was identified in 2016, besides *Ae. albopictus* [14].

To date, prevention of vector-borne diseases, such as dengue, chikungunya, and Zika fever still relies on sustainable, locally adapted, and adequate vector control, as specific antiviral treatment or vaccines for these viral diseases are still unavailable. In fact, taking yellow fever as an example, the disease continued to impose a significant burden on Africa and Latin America despite the development of an effective vaccine [18]. To maximize the efficiency of vector control programs, researchers must first establish a firm knowledge of the vectors’ ecology. Accordingly, nationwide entomological surveys were successively conducted in the DRSTP. The surveillance of mosquitoes would provide fundamental information for risk assessments, control strategies, and effect evaluation.

This report summarized mosquito surveys conducted from 2000 to 2016 in the DRSTP. The distribution of *Ae. albopictus* in the nation was illustrated, and the ecological characteristics of breeding sources as well as the genetic features of the invasive populations were delineated.

## 2. Materials and Methods

### 2.1. Ethics Statements

All mosquito specimens analyzed in the present study were collected as part of the vector surveillance and malaria control programs. Approval for this study, including mosquito collection, was granted by the Ministry of Health of the DRSTP (OF° N° 20/P° CNE/2016) and the Institutional Research Ethics Committee (NTUH-REC 201110023RD).

### 2.2. Mosquito Sampling

Entomological surveys have been conducted periodically as a part of the malaria risk assessments since 2000 in the DRSTP. Adult mosquitoes were collected using human landing catches, double-net devices, and CDC light traps nocturnally at two to four sites in each district. Local health workers participating in the actions were provided with rapid tests and pre-exposure prophylaxis for malaria [17]. The collection was performed indoors and outdoors in villages, around villages as well as in the nearby edges of forested areas. Nationwide surveys were performed in 2000, 2006, and 2015, while mosquito collections were carried out at designated sentinel sites mainly located in Agua Grande District in 2004, 2005, 2010, 2011, and 2012. Captured mosquitoes were identified morphologically [19,20]. Then, the specimens were preserved in tubes with silica gel desiccants and sent for further laboratory analyses.

An investigation specifically targeting *Aedes* mosquitoes was carried out at 15 study sites across the nation during January to February and July to September in 2015 and 2016 as a follow-up investigation performed to assess the risks of arboviral diseases after the serological findings suggested DENV exposure in the DRSTP [21] (Figure 1). Agua Grande District represents the smallest district with a dense population, while Caue and Lemba districts are relatively remote and mountainous. Sampling was performed primarily around human-domesticated environments. Adult mosquitoes were collected by sweep nets during daytime. Larvae and pupae were obtained by searching for water-filled habitats. One hundred fifty to two hundred artificial water-holding containers were visited at each study site (averaged 180 containers per site), and the types as well as the numbers of containers with *Aedes* larvae or pupae were recorded. To confirm the colonization of *Ae. albopictus*, ovitraps were placed to attract gravid females to lay eggs at fixed sites in Campo de Milho, Agua Grande, and eggs on the strips were examined weekly during the study period. Aquatic-stage mosquitoes and eggs were brought back to the insectary and reared to adulthood. Species of mosquitoes were identified according to morphologic characteristics [20,22]. Newly emerged mosquitoes with wholly morphological features were pinned as vouchers and preserved in sample boxes with mothballs in the laboratory. Specimens for molecular analysis were stored in tubes containing silica gel desiccants.

### 2.3. Molecular Analysis

Genomic DNA was extracted from whole mosquito bodies using a DNA extraction kit (Geneaid Biotech Ltd., Taipei, Taiwan) according to the manufacturer’s instructions. The universal LCO and HCO barcoding primers were used for molecular analysis [23]. The PCR mixtures consisted of 2 μL of template DNA, 1 μL of each primer at 10 μM, and 12.5 μL of HotStarTaq Master Mix (Qiagen, Hilden, Germany), and a final volume of 25 μL was obtained with ddH_2_O. DNA amplification was performed using a T3000 Thermocycler (Biometra GmbH, Jena, Germany). The following reaction condition was used: an initial incubation of 95 °C for 15 min; 35 cycles of denaturation at 94 °C for 30 s, annealing at 47 °C for 30 s, and extension at 72 °C for 40 s; followed by a final extension at 72 °C for 10 min and a 4 °C hold. The PCR products were visualized on 2% agarose gels stained with EZ-Vision^®^ DNA Dye (Amresco Inc., Solon, OH, USA) and sent for bidirectional sequencing at a commercial laboratory (Mission Biotech, Taipei, Taiwan). Completed sequences were aligned using DNASTAR Lasergene and the Clustal W algorithm implemented in BioEdit v7.2.5 software [24]. The sequences generated in the study were directly compared with those publicly available in GenBank using BLAST (http://blast.ncbi.nlm.nih.gov/, accessed on 26 October 2016). Partial mitochondrial cytochrome c oxidase subunit I (*COI*) sequences from 47 isolates of *Ae. albopictus* were aligned. The resulting alignment included 658 bases and was analyzed in MEGA11 using the Neighbor-Joining method [25].

### 2.4. PCR for Wolbachia-Specific Genes

Taking advantage of its cytoplasmic incompatibility and pathogen-blocking properties, the maternally inherited endosymbiont *Wolbachia* is being explored as a cost-effective tool for controlling arboviral transmission in many tropical countries [26,27,28]. To investigate the potential for future applications of *Wolbachia* in the DRSTP, *Wolbachia* infection in *Ae. albopictus* was screened with the *wsp* primer pair: 81F: 5′-TGGTCCAATAAGTGATGAAGAAAC-3′ and 691R: 5′-AAAAATTAAACGCTACTCCA-3′. Additional primers were used for differentiation of the infected groups, i.e., 136F/691R for group A (136F: 5′-TGAAATTTTACCTCTTT-TC-3′) and 81F/522R for group B (522R: 5′-ACCAGCTTTTGCTTGATA-3′) [29]. PCR products of individual mosquitoes were sent for sequencing (Mission Biotech, Taipei, Taiwan). The resulting sequences were compared with those in GenBank.

## 3. Results

### 3.1. Identification of Ae. albopictus during Mosquito Surveys

A total of 36 mosquito species were identified during the routine surveys conducted since 2000, of which four species, *Culex* (*Culex*) *poicilipes*, *Mansonia* (*Coquillettidia*) *annetti*, *Uranotaenia* (*Uranotaenia*) *alboabdominalis*, and *Uranotaenia* (*Uranotaenia*) *fraseri*, were newly recorded in the nation. Combined with previous records [14,16,30,31,32,33,34], a list of 44 mosquito species belonging to 11 genera has been documented in the DRSTP (Table 1). 

An investigation into *Aedes* mosquitoes with additional specimen collection during daytime was carried out in 2015, and a total of 5530 adult mosquitoes were collected. The majority belonged to *Culex* mosquitoes (3547/5530, 64.1%) which included *Culex* (*Culex*) *quinquefasciatus* (3215/5530, 58.1%), *Culex* (*Culiciomyia*) *cinerellus* (228/5530, 4.1%), *Culex* (*Culex*) *thalassius* (37/5530, 0.7%), *Culex* (*Culex*) *decens* (32/5530, 0.6%), *Culex* (*Culex*) *antennatus* (22/5530, 0.4%), *Culex* (*Eumelanomyia*) *inconspicuosus* (6/5530, 0.1%), *Culex* (*Eumelanomyia*) *micolo* (4/5530, 0.1%), *Culex* (*Culex*) *invidiosus* (2/5530, 0.04%), and *Culex* (*Culiciomyia*) *cambournaci* (1/5530, 0.02%). *Anopheles* mosquitoes were the second most abundant species (1809/5530, 32.7%) including *Anopheles* (*Cellia*) *coluzzii* (1797/5530, 32.5%) and *Anopheles coustani* (12/5530, 0.2%) (Table 2). One hundred sixty-one *Aedes* mosquitoes (161/5530, 2.9%) were found in the collection, and to our surprise, *Ae. albopictus* was identified at four sites on Sao Tome Island (119/5530, 2.2%).

### 3.2. Distribution of Ae. albopictus and its Breeding Sources

**Figure 2:**
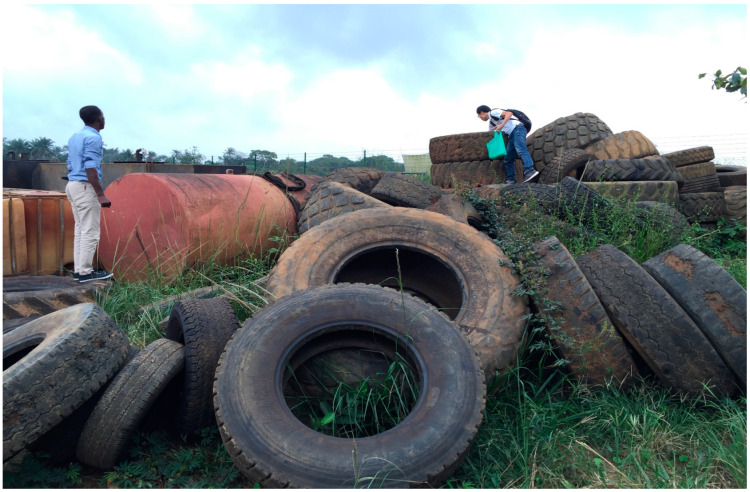
Used tires were the most common breeding source of *Ae. albopictus* in the Democratic Republic of Sao Tome and Principe. The photo shows piles of used tires near the airport on Principe Island.

Immatures of *Ae. albopictus* were discovered at all nine sentinel sites in six districts on Sao Tome Island as well as all six sites on Principe Island (Figure 1). Five species of *Aedes* larvae were observed in artificial water-holding containers in 2016, with *Ae. albopictus* being the dominant one (Table 3). Of 2698 containers with freshwater, 488 (18.1%) were positive for *Ae. albopictus* larvae or pupae. Ponte Graca in Agua Grande District had the highest positive rate for *Ae. albopictus* larvae (64/180, 35.6%) on Sao Tome Island, and the airport in Principe (Aeroporto) had the greatest overall container index for *Ae. albopictus* (114/250, 45.6%). The common types of breeding sources included used tires (*n* = 253) (Figure 2), household water-storage containers (*n* = 103), and waste cans (*n* = 65), but the larvae could also be found in natural sources, such as leaf axils and tree holes. The larvae of *Ae. albopictus* and *Ae. aegypti* co-occurred in some habitats (*n* = 7). However, *Ae. aegypti* preferred natural breeding sources to artificial containers in the DRSTP. Breeding populations of *Ae. albopictus* confirmed by eggs laid in ovitraps were found at two of six sites persistently.

### 3.3. COI-Based Molecular Analysis

**Figure 3:**
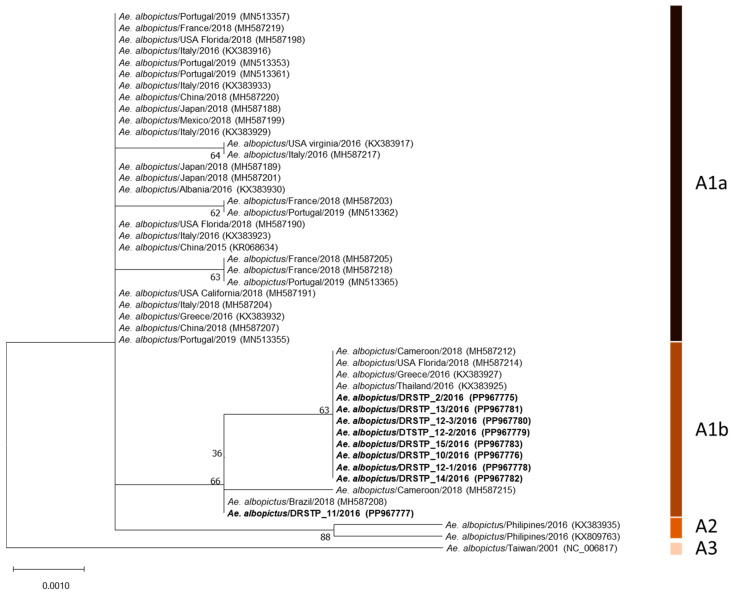
Phylogenetic analysis based on partial mitochondrial cytochrome c oxidase subunit I (*COI*) sequences of *Aedes albopictus*. The analysis was conducted in MEGA11 using the Neighbor-Joining method [25]. The evolutionary distances were computed using the Kimura two-parameter method in the bootstrap test (1000 replicates). A total of 658 positions were applied.

At least three specimens at each sampling site on Príncipe Island were subjected to molecular analysis. In total, 27 specimens of *Ae. albopictus* collected from six locations were analyzed using the *COI* gene. A very low level of polymorphism was observed among the sequences, with only one single nucleotide polymorphism (SNP) at position 2142 (G; guanosine) in the females collected at Porto Real (Figure 3). The sequence was in line with the ST3 (JF309318) identified earlier on Sao Tome Island and identical to the reference sequences from Brazil (KX383924), China (JQ235749), Democratic Republic of Congo (MT345356), India (KJ410335), Portugal (MK995332), the USA (Los Angeles, CA) (KC690940), or Vietnam (HQ398900) in GenBank [13]. Other specimens had A (adenosine) at position 2142, which was in agreement with the ST1 (JF309317) [13], and matched perfectly with the sequences from China (KU738429), Democratic Republic of Congo (MT345384), Greece (MN005056), Malaysia (MF148282), Singapore (MW321942), Thailand (KX383928), the USA (Los Angeles, CA, USA) (KC690951), or Vietnam (MZ230338).

### 3.4. Wolbachia Infection in Ae. albopictus

All 27 specimens of *Ae. albopictus*, including both males (*n* = 3) and females (*n* = 24), were superinfected by group A (*w*AlbA) and group B (*w*AlbB) *Wolbachia*. The specimens generated identical sequences of *w*AlbA (508 bp), showing 100% similarity to those from China (KU738337), India (MF805776), Mexico (KX118691), Sri Lanka (MH777434), or Thailand (KY817484). PCR targeting partial *wsp* of *w*AlbB also yielded identical sequences (400 bp) for all the specimens, which matched with sequences from China (CP041924), India (JX629463), Panama (MH392336), Singapore (MT645169), Thailand (KY817494), or the USA (St. Augustine, FL) (CP041923).

## 4. Discussion

Mosquito surveys have been routinely conducted in the DRSTP as a countermeasure of malaria control since 2000. Two novel species, *Coquillettidia saotomensis* and *Culiseta* (*Theomyia*) *wui*, were identified in 2008, and the new record of *Mimomyia* (*Etoreptiomyia*) *mediolineatafive* was demonstrated in 2009 [32,33,34]. *Culex poicilipes*, *Mn. annetti*, *Ur. alboabdominalis*, and *Ur. fraseri* were first recorded in the nation in the study. In contrast, although *Aedes africanus*, *Anopheles* (*Cellia*) *funestus*, *Anopheles* (*Anopheles*) *paludis*, *Anopheles* (*Cellia*) *pharoensis*, *Culex* (*Culex*) *pipiens*, *Culex* (*Culex*) *rima*, *Culiseta (Theomyia) fraseri*, and *Mansonia* (*Mansonioides*) *africana* were documented in early reports, the mosquitoes were not encountered in recent investigations [14,16,30,31]. The results may be affected by sampling sites, sampling time, sampling methods, seasons as well as ecological changes of the environment. Insecticide has been extensively applied for the control of malaria in the DRSTP, for example, intra-domiciliary DDT spraying campaign was implemented in 1980 to 1982, and after that, three rounds of nationwide indoor residual spraying (IRS) using pyrethroid was applied during 2004 to 2006 [35,36]. From 2007 to 2013, larvicide of *Bacillus thuringiensis israelensis* (*Bti*) was used for outdoor larval control [17]. Long-term and intensive use of insecticide has been shown to have a great impact on biodiversity. Since 1986, only *An. coluzzii* and *An. coustani* were found on the islands [37]. The influence on non-target mosquitoes was supported by the evidence of resistance to DDT in the native populations of *Ae. aegypti* [38].

In our study, inspired by the findings of antibodies against DENV in pregnant women in the nation, diurnal collections of *Aedes* mosquitoes were included in the entomological surveys since 2015 [21]. The serological evidence suggested DENV exposure and implied the existence of a transmission cycle. Thus, an investigation of the abundance of *Aedes* mosquitoes would help to assess the risk of dengue. To our surprise, *Ae. albopictus* was detected in the mosquito collections. Since its appearance in South Africa, *Ae. albopictus* has been found in Nigeria (1991), Cameroon (2000), Equatorial Guinea (2003), Gabon (2007), the Central African Republic (2009), and the Republic of Congo (2011) in the Afrotropical Region [10,39,40,41,42,43,44]. The current study identified *Ae. albopictus* during an entomological survey in 2015, but the invasion could probably be dated back as early as 2013 [15]. Our findings illustrated the nationwide distribution of the invasive populations of *Ae. albopictus*, including the densely populated plains of Agua Grande, the mountainous area of Caue, and even the autonomous region of Principe Island, which was inhabited by only 5% of the total population. Ponte Graca is a hamlet right next to the capital, Sao Tome, and it was found to have the highest positive rate of *Ae. albopictus* larvae (64/180, 35.6%) on Sao Tome Island. The positive containers were mainly water-storage tanks. However, in other less populated areas, used tires provided ideal habitats for *Ae. albopictus*, e.g., the positive rate was over 40% in Aeroporto in Principe. The difference in breeding source choices in urban and rural areas was consistent with the later observation by Kamgang, et al. (2024) [38]. Larvae of *Ae. albopictus* were also found in phytotelms. The diversity of breeding sources indeed reflected the ecological plasticity and highly invasive nature of the species.

Larvae of *Ae. albopictus* were found to coexist with *Ae. aegypti* in some habitats in the study. Larvae coexisting in the same habitats were brought back to the insectary and reared to adulthood successfully without significant inhibitory effects between species. Nevertheless, several studies have shown that *Ae. albopictus* dominated in the interspecific larval competition. The populations of native *Ae. aegypti* declined rapidly after the invasion of *Ae. albopictus*, and the later species became particularly prevalent in urban settings as well as in remote forested areas [45,46]. In our findings, larvae of *Ae. albopictus* predominated in artificial water-holding containers (488/2698, 18.1%), while other *Aedes* mosquitoes may prefer natural breeding sources. Interestingly, *Ae. aegypti* was reported to comprise 55.5% of the specimens of *Aedes* mosquitoes collected in the dry season during the entomological survey in 2022 [38]. Whether there was a rebound of *Ae. aegypti* population after regaining balance between species needed to be clarified. Two subspecies of *Ae. aegypti* with distinct morphological and ecological features exist in sub-Saharan Africa. The sylvatic *Ae. aegypti formosus* was predominant in the DRSTP in our surveys, but the subspecies has been found active sympatrically with the domestic *Ae. aegypti aegypti* in urban environments in Africa as well [47]. Previous studies further indicated that climate change and urbanization may drive *Ae. aegypti formosus* to utilize artificial water-holding containers for breeding in prolonged dry seasons and increase human biting [48]. Thus, the rise of *Ae. aegypti* could also be attributed to changes in the balance of subspecies or mosquito behaviors. In addition, details of interactions between *Ae. albopictus* and *Ae. aegypti* in the DRSTP remained to be investigated. For instance, parasitism of *Ae. albopictus* by *Ascogregarina taiwanensis* has been demonstrated to affect its competitive ability against other *Aedes* larvae [49]. There is currently a lack of research elucidating the prevalence of *Ascogregarina* spp. in *Ae. albopictus* and the native populations of *Ae. aegypti*.

A very low polymorphism in the partial *COI* gene was detected in *Ae. albopictus* collected from Principe Island. Similar results, called the founder effect, have been shown by other studies in areas newly colonized by *Ae. albopictus* [50,51]. The sequences obtained in the study, although with a different reverse primer, were consistent with the ST1 (JF309317) and ST3 (JF309318) identified in Sao Tome, while Rader, et al. (2024) described three haplotypes, ST1, Principe 1, and Principe 2, in Principe [13,15]. The two haplotypes in current study both belonged to one of the haplogroups known to associate with the recent global spread (A1b in Figure 3). The haplogroup covered the mitochondrial DNA lineages in Thailand, Malaysia, the Papua New Guinea mainland, Cameroon, and Brazil as well as at lower frequencies in California and Texas, indicating that the *Ae. albopictus* populations in the DRSTP were likely to originate from tropical regions [52,53,54]. The analysis of mitochondrial DNA could provide new insights into how the vectors spread and help to identify the paths of importation.

Geographic expansion of a vector has been shown to play a crucial role in the spread of disease [55]. *Aedes albopictus* is a capable vector for a variety of Flavivirus, Alphavirus, and Bunyavirus. The mosquito was experimentally susceptible to 36 arboviruses, and the transmission was confirmed for 14 viruses [56]. The potential threat of the mosquito was even accentuated when a mutation in the E glycoprotein (A226V) of chikungunya virus detected during the epidemic in La Reunion was demonstrated to confer improved transmission by *Ae. albopictus* [57]. Although considered the second important vector, in some cases, *Ae. albopictus* was the sole vector in the regions. Taking Europe as an example, the occurrence of chikungunya and dengue epidemics in Italy and France have just proved the public health concerns about the increased risk of arboviral diseases resulting from the invasion of *Ae. albopictus* [58,59]. In Africa, the sylvatic *Ae. aegypti* was found to be less susceptible to all four DENV serotypes, whereas studies in Gabon have demonstrated that the introduced *Ae. albopictus* exhibited relatively high vector competence for ZIKV, which was further supported by the discovery of infected *Ae. albopictus* in the field [60,61,62,63,64]. In addition, unlike the domestic *Ae. aegypti agypti*, the sylvatic *Ae. aegypti formosus* was rather zoophilic, making the introduced *Ae. albopictus* the more anthropophilic mosquito by comparison [65]. As a result, the invasive populations were held responsible for the concurrent dengue and chikungunya outbreaks in Gabon [66,67]. One limitation of the current study was the lack of information about arboviruses detected in collected *Aedes* mosquitoes. In 2022, the first dengue outbreak in the DRSTP was reported [3]. Whether the invasive *Ae. albopictus* played a role in the event and its vector competence remains to be investigated.

*Wolbachia* spp. are intracellular symbiotic bacteria that commonly infect invertebrates. Recent studies have shown that certain strains of the endosymbiont *Wolbachia* were able to lower the vectorial capacity of mosquitoes and, therefore, provided new strategies for bio-control of mosquito-borne diseases, either by directly inhibiting the infection of pathogens or by shortening mosquito lifespans [27,28]. *Aedes albopictus* has been reported naturally harboring two *Wolbachia* groups, *w*AlbA and *w*AlbB, with different prevalence [68]. The current study showed both males and females of the invasive populations of *Ae. albopictus* were superinfected by *w*AlbA and *w*AlbB. The sequences of partial *wsp* were identical to those from Southeast Asia. Artificial introduction of a third *Wolbachia* strain has been demonstrated feasible, and the resulting populations were potentially capable of blocking dengue and chikungunya transmission [69,70]. The archipelago of DRSTP is a relatively controlled environment which might serve as a promising site for pioneer field trials of releasing transfected mosquitoes. Extensive investigation into the occurrence of *Wolbachia* in vector mosquitoes in nature is essential before applying any test releases [71].

Both *Ae. aegypti* and *Ae. albopictus* are daytime biters; as a result, they would be less affected by the application of bed nets or insecticide-treated nets, a useful measure for malaria prevention. The control strategy therefore relies on source reduction to remove larvae-inhabiting containers. However, water storage is a common practice for the residents in response to a lack of piped-water supplies. Proper management of storage containers, such as using lids that are fully sealed onto the containers or applying plastic net covers to tanks, has been shown to successfully decrease larval presence [72,73]. Separately, *Bti* has been administered as an effective larvicide for malaria control in the DRSTP. Although *Anopheles* and *Aedes* mosquitoes share less similarity in breeding sources, studies have provided promising evidence for the control of *Aedes* larvae by *Bti* [74]. Considering the potential risk of arbovirus transmission by the invasive mosquito, interventions are suggested to be implemented against *Ae. albopictus*. Continual vector and disease surveillance would provide prompt epidemic information and is essential for adopting efficient control strategies.

## 5. Conclusions

Our study has provided an update of the mosquito inventory in the DRSTP. The widespread status of *Ae. albopictus* across the nation was reported with the typology of breeding sources. Sequence analysis based on the *COI* gene revealed a very low polymorphism among the invasive populations, implying the recent introduction. The potential impact of the invasion of *Ae. albopictus* on disease transmission should be taken into consideration for the implementation of vector control strategies.

## Figures and Tables

**Table 1:** Mosquito surveys conducted in the Democratic Republic of Sao Tome and Principe, 2000–2016.

Genus	Mosquito Species	Year/Period								
		2000 ^1^	2004	2005	2006 ^1^	2010	2011	2012	2015 ^1^	2016
*Aedes*	*Ae.* (*Stegomyia*) *aegypti*	+	+	+	+	+	+	+	+	+
	*Ae.* (*Stegomyia*) *albopictus*	−	−	−	−	−	−	−	+	+
	*Ae.* (*Stegomyia*) *africanus*	−	−	−	−	−	−	−	−	−
	*Ae.* (*Neomelaniconion*) *circumluteolus*	+	+	+	+	−	+	−	+	−
	*Ae.* (*Aedimorphus*) *gandarai*	−	+	+	+	−	−	−	−	−
	*Ae.* (*Aedimorphus*) *nigricephalus*	+	+	+	+	+	+	+	+	−
*Anopheles*	*An.* (*Anopheles*) *coustani*	−	+	+	+	−	+	+	+	+
	*An.* (*Cellia*) *funestus*	−	−	−	−	−	−	−	−	−
	*An.* (*Cellia*) *coluzzii*	+	+	+	+	+	+	+	+	+
	*An.* (*Anopheles*) *paludis*	−	−	−	−	−	−	−	−	−
	*An.* (*Cellia*) *pharoensis*	−	−	−	−	−	−	−	−	−
*Culex*	*Cx.* (*Culex*) *annulioris*	−	−	−	+	−	−	−	−	−
	*Cx.* (*Culex*) *antennatus*	+	+	+	+	−	+	+	+	+
	*Cx.* (*Culiciomyia*) *cambournaci*	+	+	+	+	−	−	−	+	−
	*Cx.* (*Culiciomyia*) *cinerellus*	+	+	+	+	+	+	+	+	+
	*Cx.* (*Culex*) *decens*	+	+	+	+	+	+	+	+	−
	*Cx.* (*Eumelanomyia*) *inconspicuosus*	+	+	+	+	−	+	−	+	+
	*Cx.* (*Culex*) *invidiosus*	−	+	+	+	−	+	−	+	+
	*Cx.* (*Culiciomyia*) *macfiei*	−	−	−	+	−	−	−	−	−
	*Cx.* (*Eumelanomyia*) *micolo*	−	−	−	+	−	−	+	+	−
	*Cx.* (*Culiciomyia*) *nebulosus*	−	+	−	+	−	−	−	−	−
	*Cx.* (*Culex*) *pipiens*	−	−	−	−	−	−	−	−	−
	*Cx.* (*Culex*) *poicilipes* ^3^	−	−	+	−	−	−	−	−	−
	*Cx.* (*Culex*) *quinquefasciatus*	+	+	+	+	+	+	+	+	+
	*Cx.* (*Culex*) *rima*	−	−	−	−	−	−	−	−	−
	*Cx.* (*Culex*) *tamsi*	+	−	−	+	−	−	−	−	−
	*Cx.* (*Culex*) *thalassius*	−	+	+	+	−	+	+	+	−
	*Cx.* (*Lutzia*) *tigripes*	+	+	+	+	−	+	+	+	−
*Coquillettidia*	*Cq. saotomensis* ^2^	−	−	+	−	−	−	−	−	−
*Culiseta*	*Cs.* (*Theomyia*) *fraseri*	−	−	−	−	−	−	−	−	−
	*Cs.* (*Theomyia*) *wui* ^2^	+	−	−	−	−	−	−	−	−
*Eretmapodites*	*Er. chrysogaster*	−	+	+	+	−	−	−	−	−
*Mansonia*	*Ma.* (*Mansonioides*) *africana*	−	−	−	−	−	−	−	−	−
	*Ma.* (*Coquillettidia*) *annetti* ^3^	−	−	−	+	−	−	−	−	−
*Mimomyia*	*Mi.* (*Etoreptiomyia*) *mediolineata* ^3^	+	+	+	+	+	+	+	+	−
*Polyleptiomyia*	*Po. gandarai*	−	−	−	−	−	−	−	+	+
*Toxorhynchites*	*Tx.* (*Toxorhynchites*) *brevipalpis conradti*	−	−	−	+	−	−	−	−	−
	*Tx.* (*Toxorhynchites*) *capelai*	−	+	−	−	−	−	−	−	−
*Uranotaenia*	*Ur.* (*Uranotaenia*) *alboabdominalis* ^3^	+	−	−	−	−	−	−	−	−
	*Ur.* (*Uranotaenia*) *balfouri*	+	+	+	+	+	+	+	+	+
	*Ur.* (*Pseudoficalbia*) *capelai*	−	+	+	−	−	−	−	−	−
	*Ur.* (*Uranotaenia*) *fraseri* ^3^	+	−	−	+	−	−	−	−	−
	*Ur.* (*Pseudoficalbia*) *micromelas*	−	+	+	+	−	−	−	+	+
	*Ur.* (*Pseudoficalbia*) *principiensis*	−	+	−	−	−	−	−	−	−
Total species		16	23	21	27	8	15	13	20	12

^1^ Surveys were conducted extensively in 2000, 2006, and 2015; ^2^ new species identified during our surveys; ^3^ species newly recorded during our surveys.

**Table 2:** Collection of adult mosquitoes in the Democratic Republic of Sao Tome and Principe, 2015–2016.

Mosquitoes	2015								2016							
	AG ^1^	CT ^2^	MZ ^3^	LO ^4^	LE ^5^	CU ^6^	PR ^7^	Total	AG ^1^	CT ^2^	MZ ^3^	LO ^4^	LE ^5^	CU ^6^	PR ^7^	Total
*Aedes*																
*Ae.* (*Stegomyia*) *albopictus*	56	8	34	6	0	15	0	119	14	0	0	0	0	0	0	14
*Ae.* (*Stegomyia*) *aegypti*	22	0	5	2	0	1	0	30	13	0	0	0	0	0	0	13
*Ae.* (*Aedimorphus*) *nigricephalus*	10	0	0	0	0	0	0	10	0	0	0	0	0	0	0	0
*Ae.* (*Neomelaniconion*) *circumluteolus*	2	0	0	0	0	0	0	2	0	0	0	0	0	0	0	0
*Anopheles*																
*An.* (*Cellia*) *coluzzii*	1001	148	24	194	169	203	58	1797	548	70	31	86	142	183	515	1575
*An. coustani*	12	0	0	0	0	0	0	12	7	0	0	0	0	0	0	7
*Culex*																
*Cx. antennatus*	22	0	0	0	0	0	0	22	1	0	0	0	0	0	0	1
*Cx.* (*Culiciomyia*) *cambournaci*	1	0	0	0	0	0	0	1	0	0	0	0	0	0	0	0
*Cx.* (*Culiciomyia*) *cinerellus*	170	23	0	10	2	23	0	228	3	21	1	7	2	2	0	36
*Cx. decens*	32	0	0	0	0	0	0	32	0	0	0	0	0	0	0	0
*Cx.* (*Eumelanomyia*) *inconspicuosus*	6	0	0	0	0	0	0	6	1	0	0	0	0	0	0	1
*Cx. invidiosus*	2	0	0	0	0	0	0	2	3	0	0	0	0	0	0	3
*Cx.* (*Eumelanomyia*) *micolo*	4	0	0	0	0	0	0	4	0	0	0	0	0	0	0	0
*Cx. quinquefasciatus*	3029	60	5	32	66	23	0	3215	23	0	0	0	2	0	0	25
*Cx. thalassius*	37	0	0	0	0	0	0	37	0	0	0	0	0	0	0	0
*Lutzia*																
*Lutzia* (*Metalutzia*) *tigripes*	2	0	0	0	0	0	0	2	0	0	0	0	0	0	0	0
*Mimomyia*																
*Mi.* (*Etorleptiomyia*) *mediolineata*	1	0	0	0	0	0	0	1	0	0	0	0	0	0	0	0
*Polyleptiomyia*																
*Po. gandarai*	6	0	0	0	0	0	0	6	1	0	0	0	0	0	0	1
*Uranotaenia*																
*Ur. balfouri*	3	0	0	0	0	0	0	3	3	0	0	0	0	0	0	3
*Ur.* (*Pseudoficalbia*) *micromelas*	1	0	0	0	0	0	0	1	2	0	0	0	0	0	0	2
Total	4419	239	68	244	237	265	58	5530	619	91	32	93	146	185	515	1681

^1^ Agua Grande; ^2^ Cantagalo; ^3^ MeZochi; ^4^ Lobata; ^5^ Lemba; ^6^ Caue; ^7^ Principe.

**Table 3:** Surveys of *Aedes* immatures on Sao Tome and Principe islands, 2015–2016.

Region	District	Locality	Type of Artificial Container	Containers with Larvae/Containers with Water (%)							
				2015			2016				
				Aal ^1^	Aae ^2^	Ani ^3^	Aal ^1^	Aae ^2^	Ani ^3^	Aga ^4^	Aci ^5^
Sao Tome	Agua Grande	Ponte Graca	Waste cans, water-storage tanks	20/156 (12.8)	3/156 (1.9)	1/156 (0.6)	64/180 (35.6)	12/180 (6.7)	3/180 (1.7)	6/180 (3.3)	0/180 (0)
		Compo de Milho	Discarded plastic containers, ovitraps placed at sentinel sites	16/150 (10.7)	1/150 (0.7)	3/150 (2)	45/179 (25.1)	21/179 (11.7)	0/179 (0)	0/179 (0)	0/179 (0)
		Boa Morte	Used tires, water-storage tanks	20/158 (12.7)	13/158 (8.2)	0/158 (0)	24/151 (15.9)	14/151 (9.3)	0/151 (0)	0/151 (0)	3/151 (2.0)
	Cantagalo	Santana	Used tires, discarded tools, water-storage tanks, washbasins	nd	nd	nd	22/186 (11.8)	0/186 (0)	0/186 (0)	0/186 (0)	0/186 (0)
	MeZoxi	Trindade	Waste cans	nd	nd	nd	42/181 (23.2)	0/181 (0)	0/181 (0)	2/181 (1.1)	1/181 (0)
	Lobata	Guadalupe	Used tires	7/178 (3.9)	0/178 (0)	0/178 (0)	23/186 (12.4)	0/186 (0)	0/186 (0)	0/186 (0)	0/186 (0)
	Lemba	Neves	Used tires	nd	nd	nd	45/185 (24.3)	0/185 (0)	0/185 (0)	0/185 (0)	0/185 (0)
	Caue	Sao Joao dos Angolares	Used tires	nd	nd	nd	26/197 (13.2)	2/197 (1.0)	0/197(0)	0/197 (0)	0/197 (0)
		Porto Alegre	Used tires	nd	nd	nd	6/118 (5.1)	0/118 (0)	0/118 (0)	1/118 (0.8)	0/118 (0)
Total				64/642 (10.0)	17/642 (2.6)	4/642 (0.6)	297/1563 (19.0)	49/1563 (3.1)	3/1563 (0.2)	9/1563 (0.6)	3/1563 (0.2)
Principe	Príncipe	Santo Cristo	Water-storage tanks	nd	nd	nd	13/176 (7.4)	0/176 (0)	0/176 (0)	0/176 (0)	0/176 (0)
		Porto Real	Used tires	nd	nd	nd	25/191 (13.1)	0/191 (0)	0/191 (0)	0/191 (0)	0/191 (0)
		Aeroporto	Used tires, leakage of water tower	nd	nd	nd	114/250 (45.6)	0/250 (0)	0/250 (0)	0/250 (0)	0/250 (0)
		Ponta do Sol	Waste cans, water-storage tanks	nd	nd	nd	7/146 (4.8)	0/146 (0)	0/146 (0)	0/146 (0)	0/146 (0)
		Picao	Water-storage tanks	nd	nd	nd	20/203 (9.9)	0/203 (0)	0/203 (0)	0/203 (0)	0/203 (0)
		Sao Joaquim	Buckets	nd	nd	nd	12/169 (7.1)	0/169 (0)	0/169 (0)	0/169 (0)	0/169 (0)
Total				nd	nd	nd	191/1135 (16.8)	0/1135 (0)	0/1135 (0)	0/1135 (0)	0/1135 (0)
Total				64/642 (10.0)	17/642 (2.6)	4/642 (0.6)	488/2698 (18.1)	49/2698 (1.8)	3/2698 (0.1)	9/2698 (3.3)	3/2698 (0.1)

^1^ *Aedes albopictus*; ^2^ *Aedes aegypti*; ^3^ *Aedes nigricephalus*; ^4^ *Aedes gandarai*; ^5^ *Aedes circumluteolus*. nd: not determined.

## Data Availability

All data supporting reported results can be found in context.
